# Analysis of the Process Parameters for Obtaining a Stable Electrospun Process in Different Composition Epoxy/Poly ε-Caprolactone Blends with Shape Memory Properties

**DOI:** 10.3390/polym11030475

**Published:** 2019-03-12

**Authors:** Alvaro Iregui, Lourdes Irusta, Loli Martin, Alba González

**Affiliations:** 1 POLYMAT, Department of Polymer Science and Technology, University of the Basque Country UPV-EHU, PO Box 1072, 20080 Donostia/San Sebastian, Spain; jesusalvaro.iregui@ehu.eus (A.I.); alba.gonzalez@ehu.eus (A.G.); 2 Macrobehaviour-Mesostructure-Nanotechnology SGIker Service, Polytechnic School, University of the Basque Country UPV-EHU, Plaza Europa 1, 20018 Donostia/San Sebastian, Spain; loli.martin@ahu.eus

**Keywords:** electrospinning, design of experiments, shape memory polymers, epoxy photocuring, polycaprolactone

## Abstract

In this work Poly ε-caprolactone (PCL)/ Diglycidyl ether of bisphenol A (DGEBA) blends were electrospun and the obtained mats were UV cured to achieve shape memory properties. In the majority of studies, when blends with different compositions are electrospun, the process variables such as voltage or flow rate are fixed independently of the composition and consequently the quality of the fibers is not optimized in all of the range studied. In the present work, using the design of experiments methodology, flow rate and voltage required to obtain a stable process were evaluated as responses in addition to the fiber diameter and shape memory properties. The results showed that the solution concentration and amount of PCL played an important role in the voltage and flow rate. For the shape memory properties excellent values were achieved and no composition dependence was observed. In the case of fiber diameter, similar results to previous works were observed.

## 1. Introduction

In the past 20 years, the electrospinning process [1,2,3,4,5,6] has emerged as a novel processing technique that allows the obtaining of fibers that are useful in many applications such as tissue engineering, drug delivery, composites or separation membranes [7,8,9,10,11]. More recently, the development of smart materials has allowed the obtaining of electrospun fibers with additional capabilities such as nano-fibers with self-healing properties [12] and with shape memory effect [13]. These fibers have a promising future in new emerging applications such as smart biomedical devices for minimally invasive surgery [14], self-repairing composites and textiles and intelligent packaging and membranes.

Shape memory polymers (SMP) are smart materials capable of adopting a temporary form and restoring their original shape after the application of a stimulus such as temperature, pH and UV irradiation [15,16,17]. In order to obtain a SMP, a biphasic structure material is normally used. One of the phases gives the material the definitive shape while the other provides the switching ability via thermal transitions (T_g_ or T_m_) or reversible chemical reactions [18,19,20,21,22]. Electrospinning has proven to be a good technique to obtain these kinds of SMPs as good mixing is achieved by simple solution electrospinning of a polymer blend [23], dual electrospinning [24] or coaxial electrospinning to obtain core-shell structures that present a shape memory effect [25,26] and have potential applications as smart biomedical devices and microfiber membranes.

When studying the shape memory properties of these kinds of fibers, the definitive shape material/switching material blend ratio plays an important role as it defines the sample fixing and recovery ratios. It is clear that in order to achieve good quality fibers in sufficient quantity process parameters that give rise to a stable process, such as voltage and flow rate, must be carefully selected. However, in the majority of the studies, when blends with different compositions are electrospun, the process variables are fixed independently of the composition and consequently the quality of the fibers is not optimized. According to this, the properties of the mats are affected by the poor quality of the fibers (originated by a non-appropriate selection of the process parameters) and no clear conclusions about the effect of the blend composition or other parameters on the properties can be extracted.

In a previous work [27] shape memory electrospun nano-fibers were obtained using a 50/50 wt. blend of poly ε-caprolactone (PCL) and diglycidyl ether of bisphenol A (DGEBA). The obtained mats were cured by radiation initiated cationic polymerization of epoxy groups that gave the permanent cross-linked shape to the fibers while the PCL melting gave the mats the shape changing ability. These fibers have potential applications as smart membranes and sensors. The morphology of the mats (mainly defined by fiber diameter and porosity) and the composition play an important role in the mechanical and shape memory properties of the material and, therefore, this will define their suitability for the different applications.

Taking this formulation, which presented good spinnability and shape memory properties, as a starting point in this work we are interested in evaluating the effect of the solution concentration, blend composition and photo-initiator concentration on these properties. As mentioned in previous paragraphs, in literature for carrying out these kinds of experiments electrospinning process parameters are maintained constant and therefore the quality of the fibers is not optimized [28,29,30,31], which in turn limits the analysis of the results. In order to overcome this problem, and to obtain the real composition effect, in the present work instead of maintaining the same voltage and flow rate for each composition, it was decided to adjust these parameters to obtain appropriate fiber production and a homogenous process for each solution. With the application of this novel methodology, the analyzed composition range will probably be limited because some of the compositions will not present good spinnability. However, we think that by using this approach we will avoid obtaining erroneous results that are more related with the quality of the fibers than with the studied parameters.

Therefore, we obtained fibers by changing polymer concentration, PCL/epoxy ratio and photo-initiator concentration. In order to be able to establish both the influence of these variables and the interactions between them, full factorial design (DoE) was employed to record voltage, flow rate, fiber diameter and shape memory properties as a response. Thanks to the employed methodology only good spinnability and production giving parameters were analyzed, ensuring that all the mats obtained in this work have real potential applications such as stimuli responsive sensors and membranes.

## 2. Materials and Methods

### 2.1. Materials

Bisphenol A diglycidyl ether (DGEBA, M_w_ = 340.41 g/mol), 2,2-dimethoxy-2-phenyl-acetophenone, bis(4-tert-butylphenyl)iodonium hexafluorophosphate, acetone and dimethylformamide (DMF) with analytical grade were purchased from Sigma-Aldrich (San Luis, MO, USA). Linear polycaprolactone (PCL, M_w_ = 45,000 g/mol) was purchased from Perstrop (Malmö, Sweden). All materials were used as received.

### 2.2. Electrospinning Process and Curing of the Mats

The required amount of PCL, DGEBA, the photo-initiator (bis(4-tert-butylphenyl) iodonium hexafluorophosphate) and the sensitizer (2,2-dimethoxy-2-phenyl-acetophenone) were dissolved in an acetone/DMF 3/1 weight mixture by stirring all the components required for each solution at 50 °C in a sealed container during 4 h. A homogeneous solution was obtained in each case. As other authors had previously noted, a little amount of DMF can improve the spinnability as this solvent has a higher conductivity than acetone and a lower vapour pressure [32,33]. In our case, we observed a good spinnability also employing only acetone, but the process was more stable with the addition of the DMF presenting lower clogging on the tip.

Randomly oriented fibers were electrospun by applying a voltage into a syringe loaded with the polymer solution using a high voltage source (0–30 kV, CZE1000R, Spellman High Voltage Electronics Corp, New York, NY, USA). The solution was delivered via a syringe pump (Cole-Parmer, Vernon Hills, IL, USA) and fibers were collected on a stainless steel sheet placed at 20 cm from the needle tip.

The flow rate and voltage were adjusted to ensure a drop with constant size at the end of the syringe. The minimum required voltage to start the electrospinning was measured at the same flow rate for all solutions (0.10 mL/h). This voltage was at the limit of the process, and therefore 1 kV more was employed. Once the voltage was established, the flow rate was adjusted to the minimum that allowed a constant process. Using this methodology, the solution had enough time to polarize and thin fibers without beads were obtained [34]. This protocol was used to avoid the clogging of the needle for a reasonable time and to allow the high times of deposition required to obtain reasonable mats. Mats with a thickness of around 300 microns were achieved after 8 h of electrospinning.

Environmental conditions were of 25 °C and humidity of 40%.

Electrospun mats were cured for both sides 1 h employing an UV Lamp at 365 nm (M365LP1, ThorLabs, Newton, NJ, USA) with an UV intensity of 2 mW/cm^2^.

### 2.3. Screening Design

A full factorial analysis at two levels was designed to evaluate the effect of the solution composition in diameter distribution, processing parameters, and shape memory effect. Preliminary studies were carried out to determine conditions that allowed a certain amplitude of the variables evaluated, but preserving a nanofiber type morphology, without beads. To find these values, different solutions with extreme conditions were studied: low solution concentrations with a low amount of PCL and high solution concentrations with a high amount of PCL. The criteria to select these values was to try to obtain a stable electrospinning process, achieving good fibers without beads for the first scenario and avoiding the needle clogging for the second one.

After the preliminary study, the selected variables (and levels) were the following: solution concentration (20–25 wt %), percentage of PCL (40–60%) and amount of photo-initiator (30–60 mg). Once the solutions were prepared, they were numerated and processed randomly.

JMP 13.0. (SAS Institute Inc., Cary, NC, USA) software was employed to generate a full factorial design at two levels for the three selected variables. This design of the experiment required a total of 2^3^ experiments, and the central point was repeated four times. The code names for each variable and its levels are shown in Table 1. As the response, parameters related to the process (flow rate and voltage), the final morphology (fiber diameter and its distribution) and to the shape memory effect (fixed and recovery ratio) were evaluated.

### 2.4. Characterization of Fibers

The morphology of the electrospun mats was analyzed using a Hitachi S-2700 SEM (Hitachi, Chiyoda, Japan) at 15 kV accelerating voltage, and diameter distribution was measured on 5 different images for each sample using ImageJ software (NIH, Bethesda, MD, USA) [35].

In order to characterize the curing and crystallinity degree, Fourier Transform Infrared Spectroscopy (Nicolet 6700 FTIR, Thermo Fisher Scientific, Waltham, MA, USA) equipped with a single reflection ATR system (Golden Gate, Specac, Orpington, UK), and Differential Scanning Calorimetry experiments (DSC Q2000, TA Instruments, New Castle, DE, USA) were employed. All DSC experiments were conducted at a heat rate of 20 °C/min in N_2_ atmosphere. Infrared spectra were obtained using 10 scans at a resolution of 4 cm^−1^. To calculate the crystallinity degree, fusion enthalpy was compared to the 100% crystalline PCL (135.44 J/g) taking into account the corresponding amount of PCL in the initial mixture [36].

A manual folding-deploy test was carried out to determine the shape memory properties of the mats. Three pieces of each mat were cut with dimensions of 5 × 70 mm, taking 70 mm as the initial shape (l_i_). A cylindrical glass recipient with a perimeter of 70 mm was employed as a mould. The temporary form was programmed by filling the recipient with water at 75 °C and wrapping the sample around it for 1 min. In this programmed shape, the distance between sample extremes was 0 mm ($l_{p}$). The shape fixation was accomplished by refilling the recipient with water and ice at 0 °C for 10 min to ensure the full crystallization of the PCL. After removing the recipient, the distance between extremes was measured ($l_{f}$) and considered as the fixed shape. Samples were then introduced in a water bath at 75 °C and final distance between extremes was measured and registered as the recovered shape ($l_{r}$).

Equations (1) and (2) were employed to calculate the fixity (R_f_) and recovery (R_r_) ratios:

(1) $$R_{f}\left(N\right)=\frac{l_{i}-l_{f}}{l_{i}-l_{p}}\times 100$$

(2) $$R_{r}\left(N\right)=\frac{l_{r}}{l_{i}}\times 100$$

## 3. Results and Discussion

### 3.1. Full Factorial Design

For the design of experiments, a two level full factorial design was carried out. Table 2 shows the values of the different variables of the different solutions prepared according to the levels of the design of experiment. Extreme conditions (---) and (+++) were previously studied to ensure that all the solutions were above the critical concentration and therefore they were able to be electrospun and photocured.

### 3.2. Effect of Intrinsic Variables in Electrospinning Process Parameters

The optimal electrospinning process parameters (mainly flow and voltage) to obtain a stable Taylor cone depend on the solution’s intrinsic parameters. When the design of experiments methodology is used to establish this dependence there are several approaches: to employ voltage and flow rate as selected variables [37,38], to use the same voltage and flow rate for all solutions [39,40], or to adjust these parameters in each solution. As mentioned in the introduction, we selected the last strategy in order to avoid effects due to the poor quality and low production of fibers in the response parameters. It must be taken into account that our goal was to obtain shape memory fibers and therefore, enough material to manipulate the mats was required. Therefore, not only flow rate and voltage were adjusted in each solution but also evaluated as responses in order to study how these variables were affected by the solution properties.

The voltage and flow rate employed to obtain a stable Taylor cone for each solution are shown in Table 2 and coefficients, P-values, and R^2^ for the regression model obtained for voltage are represented in Table 3. A P-value lower than 0.05 means that the factor has significance within a 95% of confidence [41].

**Figure 1:**
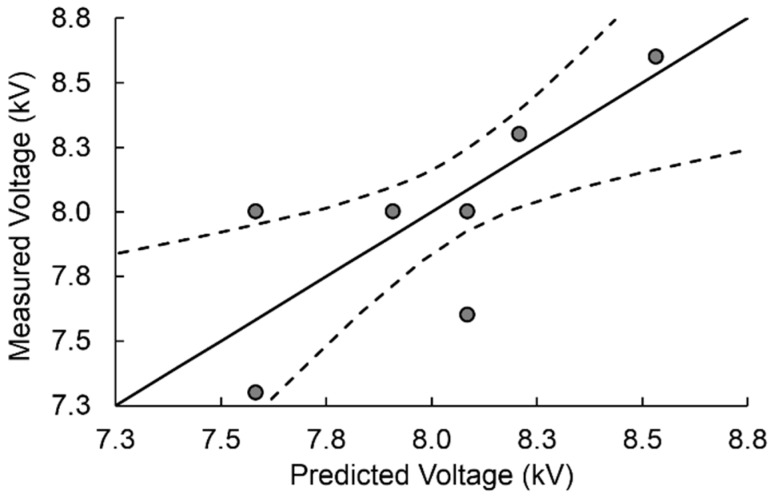
Measured vs predicted voltage, fitted multivariable line (solid) and 95% confidence curves (dotted).

Only the combined effect of X_1_ and X_2_ had a significant effect in a 95% of significance. Table 4 shows the same calculation after removing items with higher P-value. R^2^ was 0.92, so 92% of the response was explained by the model. Figure 1 shows the measured vs predicted voltage.

The combined effect of PCL percentage and solution concentration played an important role as a P-value lower than 0.01 was obtained. In our approach, the voltage was established at the same flow rate for each solution, and then flow rate was readjusted. Therefore, at the same flow rate, solutions with a higher concentration of PCL (the combined effect of X_1_·X_2_ corresponds to the total concentration of PCL in the solutions) required a higher voltage to start the process. High concentration of PCL resulted in high viscosity solutions and therefore higher voltage was required when increasing the PCL concentration [42].

In the case of X_3_, numerous authors have employed organic salts in electrospinning, and usually, the spinnability of the polymer solutions containing organic salts increases as the charge density is higher and therefore the pulling force that suffers the solution at the same voltage increases [43,44]. In our case, there was some indication that a higher amount of organic salt decreased the minimum voltage to start the process (as the coefficient for the amount of initiator was negative) but the P-value was higher than 0.05 so the confidence level was below 95%.

The final equation for the necessary voltage can therefore be extracted from the Table 4 and be expressed as:

(3) $$Voltage\;\left(kV\right)=22.6+0.013\cdot Concentration\;\left(\frac{g}{mL}\right)\cdot PCL\left(\%\right)-0.28\;\%PCL\left(\%\right)\;-0.63\cdot Concentration\left(\frac{g}{mL}\right)-0.011\cdot Initator\;\left(mg\right)$$

In the case of flow rate, values for coefficients, P-values, and R^2^ for the regression model obtained are represented in Table 5.

When all variables were studied, only X_1_ (concentration) was significant in the model. Terms related to the amount of initiator or to the combined effect of variables were high enough to be ignored. Therefore, only X_1_ and X_2_ were maintained, and the regression model was obtained again. The results of such calculation are shown in Table 6.

Considering only concentration and PCL percentage, both of them had significance inside the 95% level of confidence. The R^2^ obtained implies that only 78% of the response was explained by the model.

**Figure 2:**
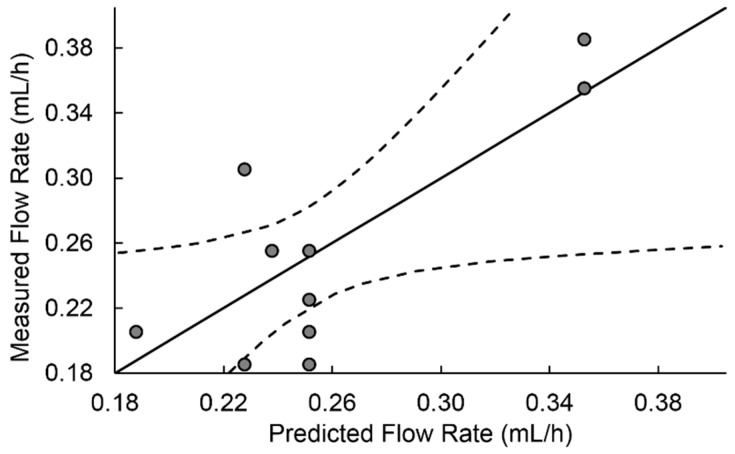
Measured vs predicted flow rate, fitted multivariable line (solid) and 95% confidence curves (dotted).

In our system, an increase of the flow rate was necessary to get a stable process when either the amount of PCL or concentration were augmented. In the case of concentration, this can be explained as a higher proportion of the drop being electrospun in the exit of the needle, and therefore to maintain the stability higher flow rate had to be applied. The same effect took place when the PCL amount was increased, as more material was electrospun (PCL was the only high molecular weight component in the system capable of being spun into fibers). Figure 2 shows the measured versus predicted flow rate values with a regression line and 95% confidence curves for the regression model employing the terms in Table 6.

The values of variables with no significance were removed, giving rise to the equation for flow rate shown below.

(4) $$Flow\;rate\;\left(\frac{mL}{h}\right)=-0.35+0.018\cdot Concentration\;\left(\frac{g}{mL}\right)+\;0.0039\cdot PCL\;\left(\%\right)$$

It must be taken into consideration that the flow rate was changed along with voltage, and that both variables were strongly dependent [45]. In a general way, a higher voltage results in a higher jet pull rate, and to obtain a uniform process high flow rates are required [37]. 

### 3.3. Curing and Thermal Characterization of the Electrospun Mats

**Figure 3:**
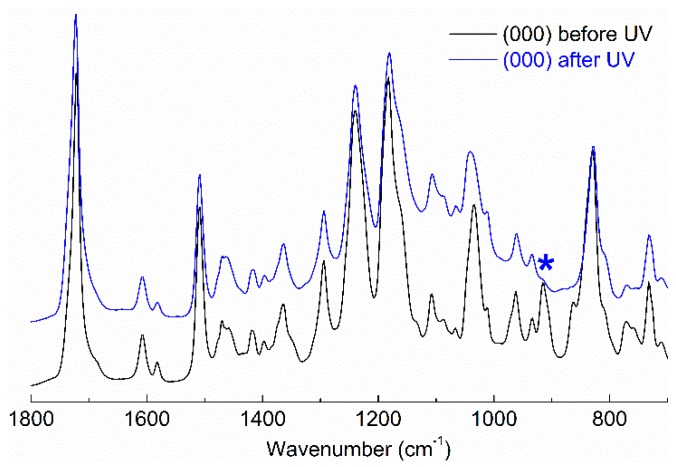
ATR spectra for a central point sample before and after curing.

After the electrospinning process, samples were separated from the collector and cured under UV irradiation. The degree of curing was determined by ATR-FTIR. Figure 3 shows the scale expanded infrared spectra of the central point of the sample before and after UV irradiation. The spectra were characterized by the bands at 1735 cm^−1^ (C=O st of the PCL), 1200–1300 cm^−1^ (C-O st of PCL and C-O-C st of epoxy ether), 910 cm^−1^ (bending of epoxy ring) and 830 cm^−1^ (=CH oop bending of epoxy aromatic ring). As can be seen in the Figure 3, the band at 910 cm^−1^ corresponding to the bending of the epoxy group disappeared after curing, indicating that the epoxy ring was opened after UV irradiation, confirming that the photopolymerization took place. All the samples displayed a similar reduction for this absorption. From the infrared spectra the conversion of the curing reaction was calculated and values higher than 95% were obtained in all the samples. As a consequence of the high curing degree the fibers maintained the shape above the melting temperature of the PCL.

**Figure 4:**
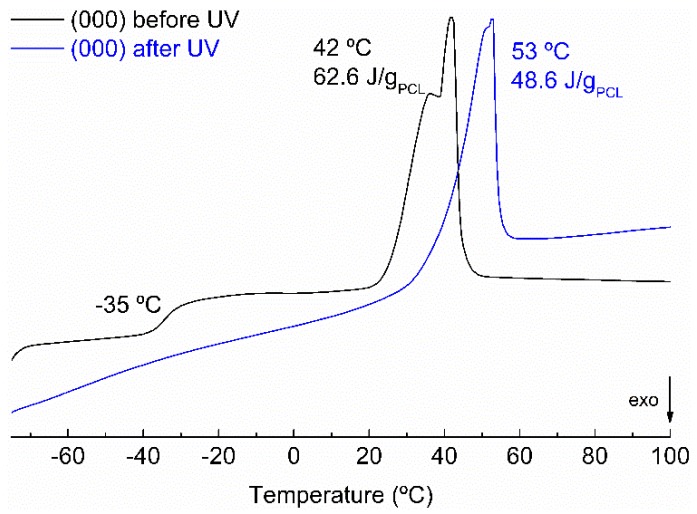
DSC thermogram (second scan) for a central point sample before and after the curing.

The melting behaviour after curing was also analyzed for all the samples by carrying out DSC experiments. Figure 4 shows the second heat scan for an electrospun sample before and after curing. Before curing, the thermogram showed a glass transition at −35 °C and a melting transition at 42 °C that could be assigned to DGEBA T_g_ and PCL melting respectively. After curing, the T_g_ of epoxy disappeared and the PCL melting temperature increased due to the fact that before irradiation DGEBA was partly soluble in the PCL, lowering the melting point. All the samples showed a similar behaviour. After curing, all the samples showed melting temperatures close to that of pure PCL and their crystallization degree was between 23 and 40%.

**Scheme 1:**
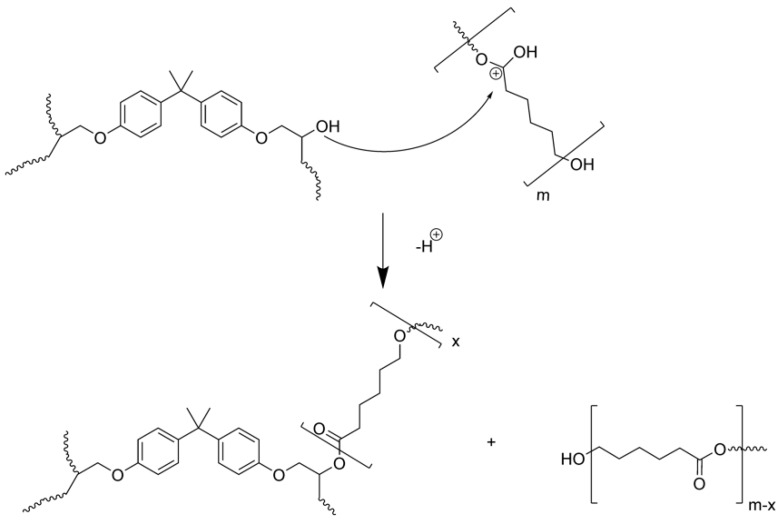
Transesterification reaction between hydroxyl groups of the epoxy and the PCL.

The final structure was determined to be a thermoset network with the PCL chemically integrated, as the gel content of the mats was around 90% (when the epoxy monomer is only up to 60%). Arnebold et al demonstrated that in the acid conditions of this kind of photopolymerization a transesterification reaction can happen [46] (Scheme 1) and this was confirmed in our system by GPC as the molecular weight of the extracted PCL was lower than the original PCL.

### 3.4. Effect of Intrinsic Variables in Diameter Distribution and Morphology of Electrospun Mats

**Figure 5:**
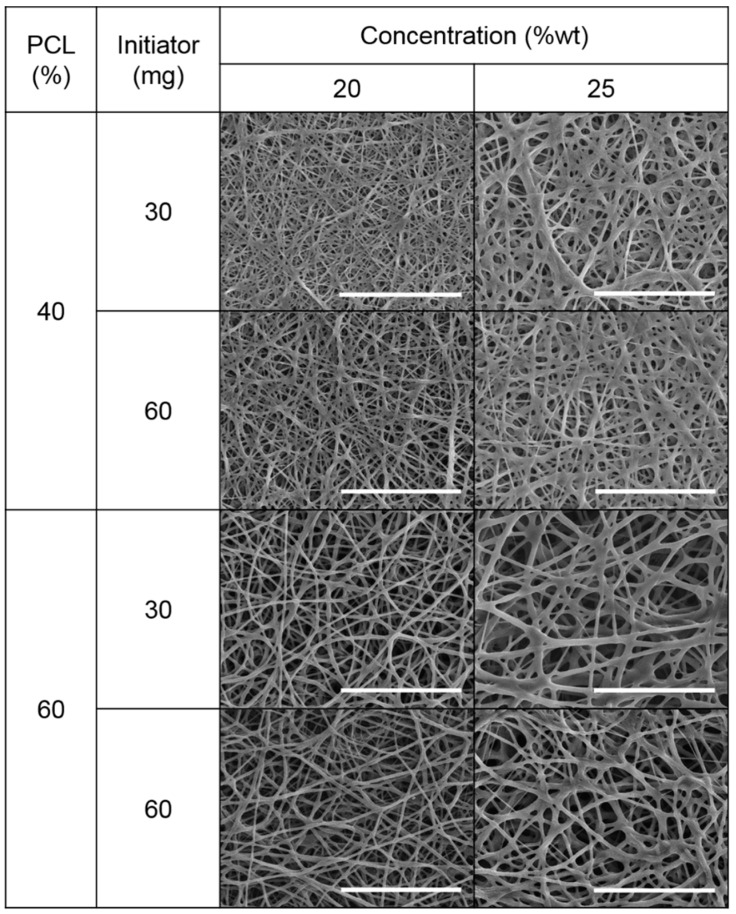
SEM images of the different mats. All scale bars represent 50 µm.

Figure 5 shows the SEM images of the electrospun mats obtained in the different experiments after the UV curing.

In all mats well-formed fibers without beads were observed. This corroborated that the selected extreme conditions were appropriate for obtaining fibers without beads. 

**Figure 6:**
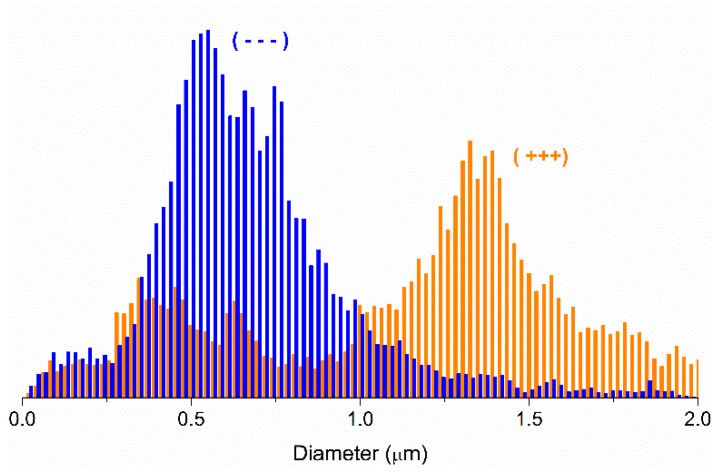
Diameter distribution for extreme conditions (- - -) and (+++).

From these images and using image J software, the fiber diameter and distribution was measured. As an example, in Figure 6 the diameter size distribution for samples (- - -) and (+++) is shown. As can be seen, for both samples the diameter did not show a normal distribution which was in accordance with previous data [27].

The mean diameter of the fibers electrospun in the different experiments is shown in Table 2. As can be seen the diameter of the fibers could be customized by changing different parameters obtaining fibers with diameters ranging from 0.70 to 1.40 μm.

In order to study the effect of the different variables in the diameter of the fiber DoE methodology was applied and the results are shown in Table 7. From the P-values in Table 7, it was clear that concentration (X_1_) played an important role in the fiber diameter, and that it was the intrinsic variable that had the greatest effect

The terms with no significance were removed and the results are shown in Table 8. As observed, the concentration (X_1_) was the main variable that defined the diameter of the fibers. As stated by many authors, and in agreement with the results obtained in this work, the diameter of the fibers increases with the polymer concentration in the electrospun solution as the amount of material capable of being spun is higher [47]. In addition, from the results of Table 2, it was clear that higher flow rates were needed to obtain fibers at higher concentrations and thus the flow rate can be also responsible for the increase observed in the diameter of the fibers. 

Fiber diameter also increased with PCL concentration (X_2_), possibly because PCL was the only high molecular weight component of the mixture. As the PCL was above the critical concentration, increasing it meant more entanglements were possible and the stretching of the fibers due to the electric force lowered and thus fibers with higher diameters were obtained [48]. In addition, at the same PCL percentage, increasing the amount of initiator (organic salt) reduced the diameter of the obtained fibers. This was also observed by other authors and can be explained by the high charge density originated by the addition of the salt. [34,47,49].

**Figure 7:**
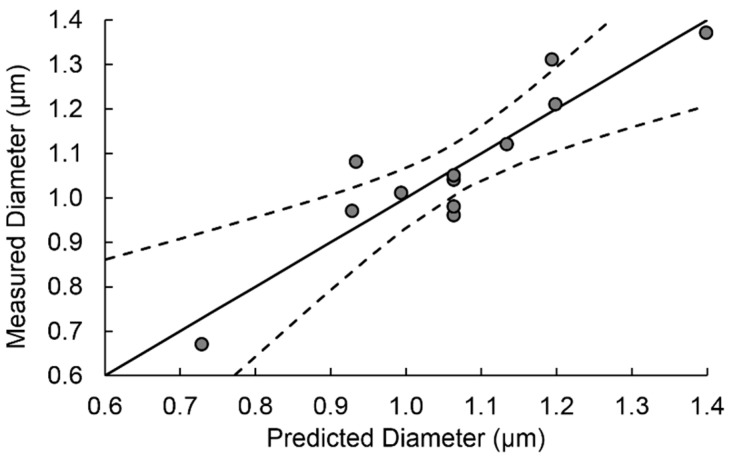
Measured vs predicted diameter, fitted multivariable line (solid) and 95% confidence curves (dotted).

It has to be noted that R^2^ was 0.984. Therefore, the measured diameter was property explained by the model. Figure 7 shows the measured vs predicted diameters obtained by the regression analysis.

The final equation for the obtained diameter can be expressed as:

(5) $$Diameter\;\left(\mu m\right)=\;-3.34+0.17\cdot Concentration\;\left(\frac{g}{mL}\right)+0.010\cdot PCL\left(\%\right)\;-0.06\cdot Initiator\left(mg\right)-0.0027\cdot Concentration\left(\frac{g}{mL}\right)\cdot Initator\;\left(mg\right)$$

### 3.5. Effect of Intrinsic Variables in Shape Memory Properties

**Figure 8:**
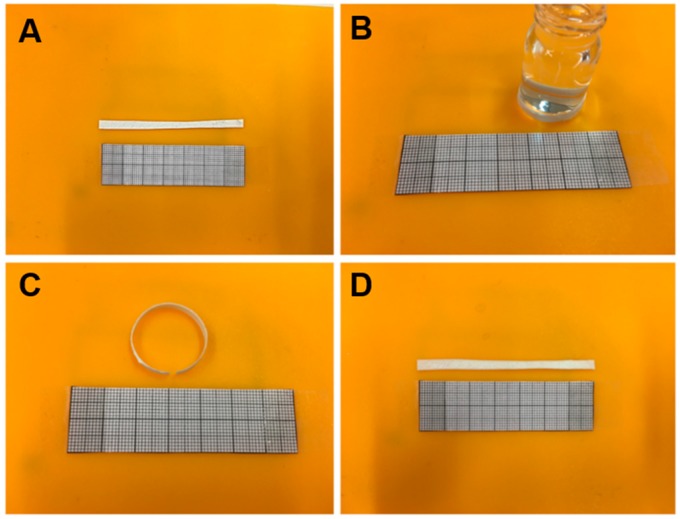
Photographs of the manual shape memory test showing (**A**) the initial longitude, (**B**) the programming shape, (**C**) the fixed shape and (**D**) the recovered sample.

The shape memory effect of the electrospun samples was measured by a manual folding-deploy test as described in Section 2.4. Figure 8 shows photographs of the process. As recorded in Table 2, all samples showed a recovery and fixity ratio between 95 and 100%, so all samples displayed excellent shape memory properties. Other authors obtained similar results in PCL based systems employing sol-gel reaction [50] or blends with other polymers such as elastomeric polyurethanes [24].

The results of the DoE analysis for the fixity and recovery ratios are shown in Table 9 and Table 10. From the results obtained in Table 9, it was clear that in the intrinsic range studied, no significant differences were obtained relating to the fixity ratio. It must be taken into consideration that the fixation process is related with the melting and crystallization of the PCL and therefore it can be argued that the concentration of this component will have an influence on the fixity ratio. However, as stated before, no influence of this variable was observed. This unexpected result can be explained taking into consideration that in the range of studied concentrations the degree of crystallinity was high enough (in DSC values ranging from 23 to 40% were obtained, as reported in the previous section) to maintain the temporary form in a good level in all the samples.

The recovery ratio was significant in a 95% level of confidence (P-value of 0.018) when only concentration was employed in the model (Table 10). However, R^2^ remained low (0.82) and therefore a great part of the response was not explained by the model. The recovery process is related to the netpoints provided by the photocuring of the epoxy resin, and therefore a relation with the epoxy and/or the photo-initiator amount were expected. However, the data did not show any dependence with these parameters probably because, as reported by FTIR analysis, the curing degree of all samples was very high.

The data showed that there was a dependency of the recovery ratio on the solution concentration. This was not easy to explain. However, it must be taken into account that when increasing the solution concentration, the diameter of the fibers increased. Therefore, it seems that fibers with higher diameter showed slightly better recovery behaviour.

## 4. Conclusions

Using a simple electrospinning process we were able to obtain customized PCL/epoxy fibers that after UV curing showed excellent shape memory properties, regardless of the level of the studied variables. The study of the electrospinning process using DoE methodology showed that in order to obtain a stable process and obtain good quality fibers, voltage and flow rate had to be adjusted for each sample as a strong relationship with solution parameters was found. The fiber diameter could be tailored by changing the solution parameters because a high dependence with solution concentration and PCL percentage was observed. All the fibers showed very good shape memory properties (fixation and recovery ratios higher than 95%). However, slightly better recovery ratios were found for the fibers obtained using high concentration that gave rise to fibers with higher diameter.

## Figures and Tables

**Table 1:** Code name and levels for each variable.

Factor	Code	Variable Levels		
		−1	0	+1
Concentration (wt %)	X_1_	20	22.5	25
PCL percentage (wt %)	X_2_	40	50	60
Initiator (mg)	X_3_	30	45	60

**Table 2:** Responses of the measured properties for each the programmed experiments.

Experiment (X_1_ X_2_ X_3_)	Voltage (kV)	Flow Rate (mL/h)	Diameter (µm)	R_f_ (%)	R_r_ (%)
(– – –)	8.6	0.20	0.67	95.2	97.6
(– – +)	8.3	0.20	0.97	99.9	98.0
(– + –)	8.0	0.30	1.08	97.3	96.8
(– + +)	8.3	0.18	1.12	95.3	96.4
(+ – –)	8.0	0.25	1.31	98.2	97.8
(+ – +)	8.0	0.25	1.01	98.8	99.2
(+ + –)	8.6	0.38	1.37	98.4	100.5
(+ + +)	8.3	0.35	1.21	97.3	100.1
(0 0 0)	8.0	0.18	0.98	101.3	95.4
(0 0 0)	8.0	0.25	1.04	97.0	97.3
(0 0 0)	7.6	0.22	0.96	99.4	96.7
(0 0 0)	8.0	0.20	1.05	97.1	97.8

**Table 3:** Analysis of variance for intrinsic properties and model coefficients for voltage.

Term		Coefficient	P-Value
Constant	C_0_	8.0583	**<0.0001**
X_1_	C_1_	0.0875	0.3369
X_2_	C_2_	−0.0875	0.3369
X_3_	C_3_	−0.1625	0.1057
X_1_X_2_	C_12_	0.3125	**0.0127**
X_2_X_3_	C_23_	0.0875	0.3369
X_1_X_3_	C_13_	−0.0875	0.3369
		F	R^2^
		1.90	0.921

Model: Voltage (y) = C_0_ + C_1_X_1_ + C_2_X_2_ + C_3_X_3_ + C_12_X_1_X_2_ + C_23_X_2_X_3_ + C_13_X_1_X_3._

**Table 4:** Analysis of variance for intrinsic properties and model coefficients for voltage after removing terms related to amount of initiator.

Term		Coefficient	P-Value
Constant	C_0_	8.0583	<0.0001
X_3_	C_3_	–0.1625	0.0873
X_1_X_2_	C_12_	0.3125	0.0050
		F	R^2^
		1.65	0.921

Model: Voltage (y) = C_0_ + C_3_X_3_ + C_12_X_1_X_2._

**Table 5:** Analysis of variance for intrinsic properties and model coefficients for flow rate.

Term		Coefficient	P-Value
Constant	C_0_	0.24667	<0.0001
X_1_	C_1_	0.04375	0.0440
X_2_	C_2_	0.03875	0.0639
X_3_	C_3_	–0.01875	0.3033
X_1_X_2_	C_12_	0.01875	0.3033
X_2_X_3_	C_23_	–0.01875	0.3033
X_1_X_3_	C_13_	0.01125	0.5220
		F	R^2^
		4.50	0.944

Model: Flow rate (y) = C_0_ + C_1_X_1_ + C_2_X_2_ + C_3_X_3_ + C_12_X_1_X_2_ + C_23_X_2_X_3_ + C_13_X_1_X_3_.

**Table 6:** Analysis of variance for intrinsic properties and model coefficients for flow rate after removing terms related to amount of initiator.

Term		Coefficient	P-Value
Constant	C_0_	0.2467	<0.0001
X_1_	C_1_	0.0438	0.0280
X_2_	C_2_	0.0388	0.0457
		F	R^2^
		4.75	0.783

Model: Flow rate (y) = C_0_ + C_1_X_1_ + C_2_X_2_.

**Table 7:** Analysis of variance for intrinsic properties and model coefficients for fiber diameter.

Term		Coefficient	P-Value
Constant	C_0_	1.0642	<0.0001
X_1_	C_1_	0.1325	0.0109
X_2_	C_2_	0.1025	0.0284
X_3_	C_3_	–0.0150	0.6739
X_1_X_2_	C_12_	–0.0375	0.3151
X_2_X_3_	C_23_	–0.1000	0.0309
X_1_X_3_	C_13_	–0.0150	0.6739
		F	R^2^
		10.03	0.984

Model: Diameter (y) =C_0_ + C_1_X_1_ + C_2_X_2_ + C_3_X_3_ + C_12_X_1_X_2_ + C_23_X_2_X_3_ + C_13_X_1_X_3_.

**Table 8:** Analysis of variance for intrinsic properties and model coefficients for fiber diameter after removing terms with no significance.

Term		Coefficient	P-Value
Constant	C_0_	1.0642	<0.0001
X_1_	C_1_	0.1325	0.0025
X_2_	C_2_	0.1025	0.0101
X_1_X_3_	C_13_	–0.1000	0.0114
			
		F	R^2^
		5.53	0.984

Model: Diameter (y) = C_0_ + C_1_X_1_ + C_2_X_2_ + C_13_X_1_X_3_.

**Table 9:** Analysis of variance for intrinsic properties and model coefficients for fixity ratio.

Term		Coefficient	P-Value
Constant	C_0_	97.934	<0.0001
X_1_	C_1_	0.625	0.4101
X_2_	C_2_	−0.475	0.5250
X_3_	C_3_	0.275	0.7089
X_1_X_2_	C_12_	0.150	0.8378
X_2_X_3_	C_23_	−0.400	0.5901
X_1_X_3_	C_13_	−1.050	0.1915
		F	R^2^
		0.79	0.639

Model: Fixity ratio (y) = C_0_ + C_1_X_1_ + C_2_X_2_ + C_3_X_3_ + C_12_X_1_X_2_ + C_23_X_2_X_3_ + C_13_X_1_X_3._

**Table 10:** Analysis of variance for intrinsic properties and model coefficients for recovery ratio.

Term		Coefficient	P-Value
Constant	C_0_	97.800	<0.0001
X_1_	C_1_	1.100	0.0719
X_2_	C_2_	0.150	0.7688
X_3_	C_3_	0.125	0.8062
X_1_X_2_	C_12_	0.750	0.1814
X_2_X_3_	C_23_	0.125	0.8062
X_1_X_3_	C_13_	–0.325	0.5311
		F	R^2^
		2.85	0.871

Model: Recovery ratio (y) = C_0_ + C_1_X_1_ + C_2_X_2_ + C_3_X_3_ + C_12_X_1_X_2_ + C_23_X_2_X_3_ + C_13_X_1_X_3._
